# Colorimetric Detection of Urease-Producing Microbes Using an Ammonia-Responsive Flexible Film Sensor

**DOI:** 10.3390/bios12100886

**Published:** 2022-10-17

**Authors:** Yunsoo Chang, Tae-Eon Park, Seung-Woo Lee, Eun-Hee Lee

**Affiliations:** 1 Department of Microbiology, Pusan National University, Busan 46241, Korea; 2 Center for Spintronics, Korea Institute of Science and Technology, Seoul 02792, Korea; 3 Department of Fine Chemistry, Seoul National University of Science and Technology, Seoul 01811, Korea; 4 Center for Functional Biomaterials, Seoul National University of Science and Technology, Seoul 01811, Korea

**Keywords:** urease, ureolytic microorganism, colorimetry, pH-sensitive, film, reusable sensor

## Abstract

Urease-producing (ureolytic) microbes have given rise to environmental and public health concerns because they are thought to contribute to emissions of ammonia and to be a virulence factor for infections. Therefore, it is highly important to have the ability to detect such microbes. In this study, a poly(dimethylsiloxane) (PDMS)-based colorimetric film sensor was employed for the detection of urease-producing microbes. The sensor was able to detect the enzyme activity of commercially available urease, as the color and absorbance spectrum of the sensor was observed to change upon being exposed to the reaction catalyzed by urease. The ratio of the absorbance of the sensor at 640 nm to that at 460 nm (A_640_/A_460_) was linearly proportional to the amount of urease present. The performance of the sensor was validated by the results of a sensitivity and selectivity analysis towards thirteen different bacterial strains. Based on the development of blue color of the sensor, the tested bacteria were classified as strongly positive, moderately positive, weakly positive, or negative urease producers. The response of the sensor to ureolytic bacteria was verified using the urease inhibitor phenyl phosphorodiamidate (PPDA). Additionally, the sensor achieved the selective detection of ureolytic bacteria even in the presence of non-ureolytic bacteria. In addition, a used sensor could be reverted to its original state by being subjected to simple aeration, and in this way the same sensor could be used at least five times for the detection of bacterial urease activity.

## 1. Introduction

**Figure 1:**
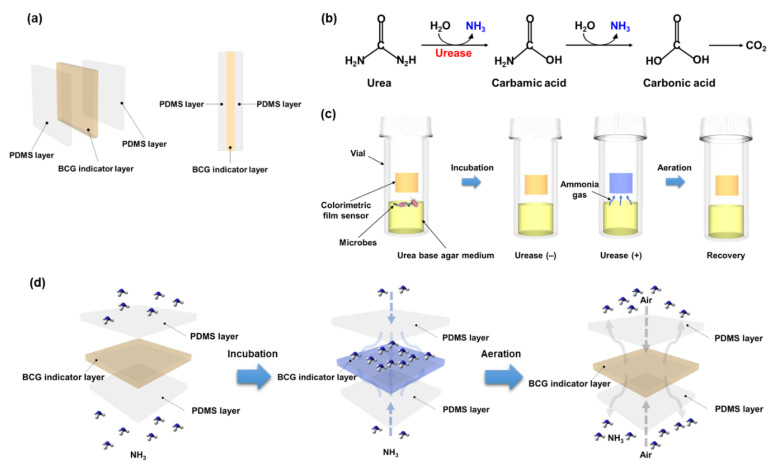
Schematic diagram of the colorimetric detection of microbial urease activity using the designed film sensor. (**a**) Composition of the colorimetric film sensor. (**b**) Reaction mechanism of the enzymatically catalyzed hydrolysis of urea. (**c**) Schematic of the experimental procedure used for the detection of ureolytic bacteria. (**d**) Reversible changes in the color of the sensor upon its exposure to ammonia gas and subsequent aeration.

Urease (urea amidohydrolase EC 3.5.1.5) is ubiquitously found in plants, fungi, bacteria, algae, and even invertebrates [1,2], where it performs basic biological functions, but it also has widespread implications for the environment, society, and human health due to its catalyzing the consumption of urea and hence causing the production of ammonia. Specifically, this nickel-dependent metalloenzyme catalyzes the hydrolysis of urea (CO(NH_2_)_2_) to ammonia (NH_3_) and carbamic acid (H_2_NCOOH), which itself undergoes a spontaneous hydrolytic decomposition to carbonic acid (H_2_CO_3_) and another molecule of ammonia (Figure 1) [2,3,4,5]. As a result of its catalyzing the production of ammonia, urease increases the pH of the surrounding environment.

Urease-producing (ureolytic) microbes are present in various settings. For example, they are found in the soil and water environments. But since urea is a source of organic nitrogen (N) and hence is important for soil fertility (note, for example, that urea-based fertilizers as a nitrogen source accounted for 55% of synthetic fertilizers globally in 2014 [6]), the transformation of urea to ammonia gas due to the actions of ureolytic microbes results in ammonia emissions and in a loss of nitrogen fertilizer for agriculture and has thus created environmental and societal concerns [6].

Perhaps even more importantly, ureolytic microbes are also present in the bodies of humans and other animals, and various pathogenic microbes produce urease in order to utilize urea as a nitrogen source [7]. Urea is the major nitrogenous waste product of most terrestrial animals, including humans; thus, bacteria colonized in human bodies exploit, by hydrolyzing urea, the resulting ammonium product as a nitrogen source [8]. Moreover, urease helps human pathogens, including *Helicobacter pylori*, *Yersinia enterocolitica*, and *Proteus mirabilis*, adapt to acidic environments [7,9,10,11,12]. The priority pathogen list of the World Health Organization (WHO) includes urease-producing bacteria, and these bacteria utilize urease as a virulence factor to infect and colonize the host [1,2,7,13]. Therefore, urease-producing microbes are of great interest due to the relevance of their enzymatic activity in infections [7,8,14,15].

Urease activity can be measured using commercially available kits, such as the urease activity assay kit produced by Sigma-Aldrich (Saint Louis, MO, USA) and a specifically ammonia assay kit produced by Abcam (Cambridge, UK). These assays, based on the Berthelot method, form a colored product—so a positive response can be detected colorimetrically [16]. The kits are easy to use and provide a sensitive performance for the detection of urease activity but often require sample preparation steps, such as filtration or deproteination prior to testing. In addition, the rapid urease test, also known as the *Campylobacter*-like organism (CLO) test, is used for providing a diagnosis of *Helicobacter pylori* infection [17,18]. The test depends on pH, so an increase in the pH in the medium caused by urease from *Helicobacter pylori* induces a color change of the specimen, specifically from yellow to red. The test allows a simple and rapid diagnosis of *Helicobacter pylori* [17]. However, the color change is influenced by the pH of the sample environment, so false-negative or false-positive results sometimes occur [19,20].

Urease-producing microbes have also been detected using a pH-independent colorimetric assay [21]; specifically, Santopolo et al. employed gold nanoparticles (AuNPs) for the color signal generation, with AuNP aggregation prevented in the presence of urease and showing a red color but allowed in its absence and showing a blue color, providing a sensitive detection of urease-positive bacteria [21].

In this study, we deployed a three-layered colorimetric film sensor for the detection of ureolytic microbes. The sensor included poly(dimethylsiloxane) (PDMS) and was shown to be responsive to ammonia gas [22], changing its color from yellow to blue due to the reaction catalyzed by urease. The colorimetric signal of the sensor was measured using commercially available urease. The performance of the sensor for the detection of urease activity was validated by carrying out a sensitivity and selectivity analysis against thirteen different microorganisms. Based on their pathogenic potential, thirteen bacteria were selected as model microbes in this study. Urease-producing microbes were identified by the observed changes in the color and absorbance spectrum of the sensor. Finally, the reversibility and reproducibility of the sensor was investigated to determine its amenability to repeated use.

## 2. Materials and Methods

### 2.1. Preparation of a Colorimetric Three-Layered Film Sensor

The colorimetric film sensor consisted of a bromocresol green (BCG)-incorporated PDMS sensing layer, which was sandwiched by the top and bottom protection films of PDMS (Figure 1a), as reported in our previous study [22]. Briefly, PDMS and its curing agent (K1 solution, Gwangmyeong-si, Gyeonggi-do, Korea) (10:1, *w*/*w*) were first mixed with a spatula. The pre-mixed PDMS was degassed in a plastic desiccator connected to a vacuum pump (Edwards Ltd., Burgess Hill, UK) to eliminate bubbles. The degassed PDMS of 0.5 g was carefully transferred to a 90-mm-diameter Petri dish plate and spread out on the dish using a spin coater (ACE-200, Dong Ah Trade Co., Seoul, Korea) at 6000 rpm for 15 s. The bottom PDMS layer was fully cured at 70 °C on a hot plate (PC-420D, Corning, New York, NY, USA) for 1 h. The sensing BCG-PDMS layer was prepared by mixing bromocresol green sodium salt (BCG, Sigma-Aldrich) and the pre-mixed PDMS. One hundred microliters (100 μL) of a BCG solution (37.5 mg/mL in deionized (DI) water) was added to 1 g of the pre-mixed PDMS to be a desired BCG concentration of 5 mM. The BCG-PDMS mixture was homogenized by a magnetic stirrer. Bubbles formed during the mixing process were removed using a vacuum pump. The BCG-PDMS mixture of 0.5 g was transferred onto the cured bottom PDMS layer on the Petri dish and spread out at 3000 rpm for 15 s using a spin coater. The same curing process as described above was introduced to form a BCG-PDMS/PDMS layer. Finally, the top layer of PDMS was fabricated on the BCG-PDMS/PDMS layer through the spin-casting of a pre-mixed PDMS of 0.5 g. The spin-casting and curing conditions were identical to the process employed for the fabrication of the bottom PDMS layer. The fully cured PDMS/BCG-PDMS/PDMS film was cut into 1 cm × 1 cm pieces and used for the colorimetric detection of urease activity.

### 2.2. Preparation of Urea Base Agar Media

Urea base (0.5 g peptone (Sigma-Aldrich), 0.5 g dextrose (Sigma-Aldrich), 2.5 g sodium chloride (Samchun Chemicals Co. Ltd., Seoul, Korea), 1 g potassium phosphate monobasic (Sigma-Aldrich), and 10 g urea (Sigma-Aldrich)) was dissolved in 50 mL of DI water, and the resulting mixture was filtered using a syringe filter (0.45 μm, Whatman, Maidstone, UK). Additionally, a mass of 10 g of agar (BD, Franklin Lakes, NJ, USA) was dissolved in 450 mL of DI water, and the resulting solution was autoclaved (MDM-60ST, MDM, Suwon-si, Gyeonggi-do, Korea) at 121 °C for 15 min and then allowed to cool down to 50–55 °C. At this point, the urea base solution was added to the agar solution and this composition was gently mixed. Six milliliters (6 mL) of the resulting liquid mixture were added into respective 20-mL glass vials and, at ambient temperature, allowed to solidify for 40 min. The vials were then closed using screw caps and stored at 4 °C prior to use.

### 2.3. Detection of Urease Enzyme Activity

**Figure 2:**
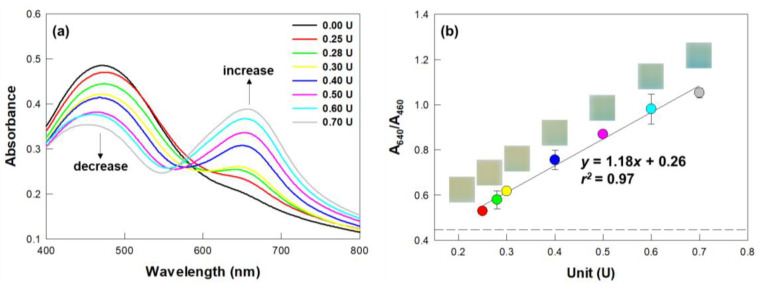
Detection of urease enzyme using the colorimetric film sensor. (**a**) Absorbance spectra and (**b**) A_640_/A_460_ values of sensors exposed to various units of urease. The dashed line depicts the A_640_/A_460_ value of the sensor not exposed to urease (0.00 U, negative control). Symbols and error bars indicate the mean and standard deviations of biological triplicates. Insets show photographs of the sensors exposed to the various units of urease.

For each experiment, a piece of the above-described colorimetric film sensor was attached to the inside of the 20-mL glass vial, specifically with the bottom of the piece of the sensor ~0.5 cm above the top of the urea base agar medium (Figure 1c). The enzyme urease (Type III, powder, Sigma-Aldrich) was dissolved in DI water, and 10 μL of this solution was loaded onto the surface of a urea medium placed in a vial as described above. The final amounts of added urease were 0.25, 0.28, 0.30, 0.40, 0.50, 0.60, and 0.70 U. A volume of 10 µL of DI water was added into another vial as a negative control (marked as 0.00 U in Figure 2). The vials were tightly capped and incubated (VS-8480MX-04-DT, Visionbionex, Bucheon-si, Gyeonggi-do, Korea) at 30 °C for 4 h. After a given incubation, the sensors were photographed and subsequently detached from the vials and transferred to cuvettes, from which absorbance spectra of the sensors were acquired using a UV spectrophotometer (UV-1800, Shimadzu, Kyoto, Japan) at a wavelength range of 400–800 nm. For each spectrum, the ratio of the absorbance at 640 nm to that at 460 nm (A_640_/A_460_) was used to determine the amount of urease. The experiments were performed in triplicate.

### 2.4. Colorimetric Detection of Ureolytic Bacteria Using the Produced Sensor

*Escherichia coli* (*E. coli*) was purchased from the Korean Collection for Type Cultures (https://kctc.kribb.re.kr/, (accessed on 7 October 2022), Jeongeup-si, Jeollabuk-do, Korea, Table 1). Twelve different bacterial strains were obtained from Nakdonggang National Institute of Biological Resources (https://fbp.nnibr.re.kr/fbcc/, (accessed on 7 October 2022), Sangju-si, Gyeongsangbuk-do, Korea, Table 1). All of the bacteria were cultured in a nutrient broth (MBcell, KisanBio Co., Seoul, Korea) at 30 °C with shaking at 180 rpm (VS-8480MX-04-DT, Visionbionex) for 24 h. In order to collect cells, the culture broth was centrifuged at 5000 rpm (Avanti j-e, Beckman Coulter, Brea, CA, USA) for 10 min and resuspended with fresh nutrient broth to a cell concentration of 1.5 ± 0.4 × 10^8^ − 2.2 ± 0.2 × 10^9^ colony-forming units per milliliter (CFU/mL) (Table 1). It is noting that the pH of the resuspended culture broth of bacteria was in a range of 7.37–7.45. Ten microliters (10 μL) of each culture broth were separately added into the vials containing a sensor and urea agar medium. The vials containing the inoculated media were incubated at 30 °C in static conditions. The sensors were periodically sampled during the incubation. The sampled sensors were photographed, and their absorbance spectra were acquired using UV–Vis spectrometry as described above. The time it took for the ammonia-producing reaction to occur was determined based on how long it took for the sensor to turn blue as determined visually. The experiments were carried out in triplicate.

### 2.5. Verification of the Performance of the Sensor

The preculture of *Klebsiella pneumoniae* (*K. pneumoniae*) broth was collected by subjecting the broth to centrifugation at 5000 rpm for 10 min and resuspended with fresh nutrient broth to produce a cell concentration of 4.2 ± 2.0 × 10^8^ CFU/mL. A volume of ninety microliters (90 μL) of *K. pneumoniae* culture broth was mixed with DI water to produce a total volume of 100 μL. Ten microliters (10 μL) of the culture solution (referred to as non-treated cells) were added into vials containing film sensors and urea agar media. At the same time, a volume of 90 μL of *K. pneumoniae* culture was treated with 10 μL of a solution of 0.1 mM phenyl phosphorodiamidate (PPDA, Alfa Aesar, Ward Hill, MA, USA) in DI water. Ten microliters (10 μL) of the PPDA-treated *K. pneumoniae* cells (referred to as PPDA-treated cells) were separately added into other vials containing sensors and urea agar media. The vials were subjected to static incubation at 30 °C. The sensors were analyzed at incubation durations of 0.0, 2.0, 5.0, 6.0, 7.5, 8.0, 10.0, and 11.0 h; specifically, they were photographed and transferred to cuvettes, from which their absorbance spectra were acquired in the wavelength range of 400–800 nm. All of the experiments were performed in triplicate.

### 2.6. Effects of Non-Ureolytic Bacteria on the Colorimetric Detection of the Urease Activity of Klebsiella Pneumoniae

Aliquots (3.3 µL) of *K. pneumoniae* culture with a cell concentration of 4.2 ± 2.0 × 10^8^ CFU/mL were added to the vials containing film sensors and urea agar media. Subsequently, the aliquots of each non-ureolytic bacterium were added to separate vials: vial labelled A, *Citrobacter koseri* (*C. koseri*, 2.2 ± 0.1 × 10^9^ CFU/mL) + *E. coli* (9.3 ± 4.0 × 10^8^ CFU/mL) + *Klebsiella michiganensis* (*K. michiganensis*, 6.2 ± 1.0 × 10^8^ CFU/mL) + *Providencia alcalifaciens* (*P. alcalifaciens*, 2.2 ± 0.2 × 10^9^ CFU/mL) (non-ureolytic bacteria only, negative control); B, *K. pneumoniae* only (4.2 ± 2.0 × 10^8^ CFU/mL) (positive control); C, *K. pneumoniae* (4.2 ± 2.0 × 10^8^ CFU/mL) + *C. koseri* (2.2 ± 0.1 × 10^9^ CFU/mL); D, *K. pneumoniae* (4.2 ± 2.0 × 10^8^ CFU/mL) + *E. coli* (9.3 ± 4.0 × 10^8^ CFU/mL); E, *K. pneumoniae* (4.2 ± 2.0 × 10^8^ CFU/mL) + *K. michiganensis* (6.2 ± 1.0 × 10^8^ CFU/mL); F, *K. pneumoniae* (4.2 ± 2.0 × 10^8^ CFU/mL) + *P. alcalifaciens* (2.2 ± 0.2 × 10^9^ CFU/mL); and G, *K. pneumoniae* (4.2 ± 2.0 × 10^8^ CFU/mL) + *C. koseri* (2.2 ± 0.1 × 10^9^ CFU/mL) + *E. coli* (9.3 ± 4.0 × 10^8^ CFU/mL) + *K. michiganensis* (6.2 ± 1.0 × 10^8^ CFU/mL) + *P. alcalifaciens* (2.2 ± 0.2 × 10^9^ CFU/mL). The vials were incubated at 30 °C for 11 h. After a given incubation time, the sensors were photographed and, for each case, A_640_/A_460_ was measured to determine the activity of urease. All of the experiments were carried out in triplicate.

### 2.7. Reusability of the Sensor

For each experiment, 10 µL of *Proteus terrae* (*P. terrae*) culture with a cell concentration of 1.4 ± 0.2 × 10^9^ CFU/mL were added to a vial containing the colorimetric film sensor and urea agar medium. The vial was then incubated at 30 °C for 4 h, after which the sensor was photographed and its A_640_/A_460_ value was measured to determine the activity of urease. After these measurements, the sensor was aerated for 2 d at ambient temperature and its absorbances at 460 and 640 nm were measured again to determine the ability to reuse the sensor. The aerated sensor was reused for the detection of urease activity of *P. terrae* cells in the same manner as described above. The sensor was used five times. The experiments were performed in triplicate.

## 3. Results and Discussion

### 3.1. Design of the Colorimetric Film Sensor for the Detection of Ureolytic Microbes

A colorimetric film sensor was employed for the detection of ureolytic activity in microbes (Figure 1). The sensor consisted of three functional layers, all containing flexible PDMS elastomer (Figure 1a). In the middle layer of the sensor, a BCG indicator was incorporated into the PDMS elastomer. Note that BCG is a pH indicator that exhibits a significant colorimetric response, from yellow to blue, according to the pH level: at pH levels between 3 and 4, BCG exhibits a yellow color; as the pH is increased to 5 to 8, the BCG is deprotonated by basic substances, resulting in a change in its structure from a monoanionic form to a dianionic form, and this change in structure leads to the change in color from yellow to blue [22]. This designed sensing layer (i.e., the BCG-PDMS layer), with a thickness of ~60 µm, was embedded between the top and bottom of the hydrophobic thin layers of PDMS each with a thickness of ~20 µm. Gaseous basic molecules can pass through PDMS [23,24,25]. On the other hand, water cannot penetrate the PDMS of the sensor, and thus false-positive results caused by alkaline solutions are minimized. Our previous study demonstrated that the sensor was able to selectively detect gaseous ammonia over various other basic substances (specifically, NaOH, KOH, and Ba(OH)_2_) [22].

Urease catalyzes the hydrolysis of urea to ammonia and carbon dioxide (Figure 1b). Therefore, the amount of ammonia increased during the enzymatic hydrolysis of urea by ureolytic (i.e., urease-positive (+)) microbes and, accordingly, the color of the sensor changed from yellow to blue (Figure 1c). On the other hand, the color of the film did not change if the microbes do not have urease activity (i.e., are urease-negative (−)) (Figure 1c). In this regard, ureolytic microbes were simply detected using our sensor and the colorimetric response was easily identified by the naked eye.

This change in the color of the sensor can be reversed upon its exposure to the fume of acetic acid or aeration [22]. As a result, the original yellow color of the film was regenerated (Figure 1d). The reversibility of the change in color of the film sensor makes it reusable, which reduces detection expenses. In addition, access to skilled technicians and expensive instruments is unnecessary for this detection of ureolytic microbes. The simplicity and reusability of the film sensor makes it affordable.

### 3.2. Validation of the Sensor for the Detection of Urease

The performance of the colorimetric film sensor was validated using a commercially available urease enzyme (Figure 2). The absorbance spectrum of the sensor changed upon the addition of urease (Figure 2a). Specifically, as the amount of urease was increased from 0.25 to 0.70 U, the absorbance at 460 nm (A_460_) decreased and the absorbance at 640 nm (A_640_) concomitantly increased. The A_640_/A_460_ value was found to be linearly proportional to the amount of urease, with the regression equation *y* = 1.18 *x* + 0.26 and *r^2^* = 0.97 (Figure 2b). The color of the sensor changed from yellow to blue upon it being exposed to the products of the reaction catalyzed by urease and the blue color became distinct in a manner dependent on the amount of urease. The development of blue color by the sensor was clearly identified by the naked eye. The results demonstrated that our pH-sensitive sensor can be used to visually detect urease activity by showing changes in its color (i.e., from yellow to blue) as well as in its absorbance spectrum upon the urease-catalyzed hydrolysis of urea [6].

### 3.3. Colorimetric Detection of Ureolytic Microbes using the Sensor

**Figure 3:**
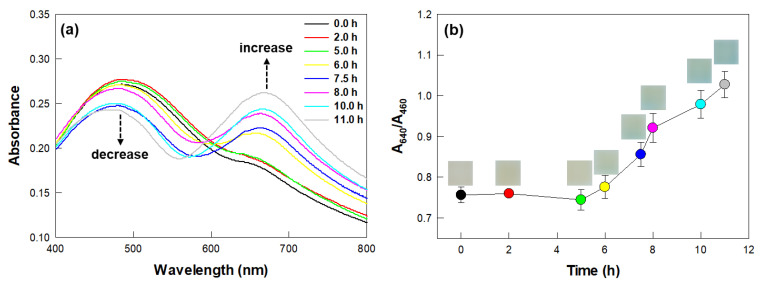
Colorimetric detection of ureolytic activity in *Klebsiella pneumoniae* (*K. pneumoniae*). (**a**) Absorbance spectra and (**b**) A_640_/A_460_ values of the colorimetric film sensors exposed to *K. pneumoniae* for the indicated incubation times. Symbols and error bars indicate the mean and standard deviations of biological triplicates. Insets show photographs of film sensors at each incubation time.

Urease-producing microbes were detected using the colorimetric film sensor. Of the thirteen tested bacterial strains, nine of them showed urease activity and different reaction times (Table 1). The sample of the bacterium *K. pneumoniae* was indicated to be positive for urease, according to changes of the absorbance spectrum of the film sensor as the incubation duration was increased (Figure 3a). Specifically, the sensor exhibited a decrease in absorbance at a wavelength of 460 nm and an increase at 640 nm, which corresponded to the results for the detection of commercially available urease enzymes, as shown in Figure 2a. The color of the sensor began to change from yellow to blue at 6 h of incubation, with the change attributed to the deprotonation of BCG in PDMS, resulting from the hydrolysis of urea by the urease-producing microbes. Accordingly, the A_640_/A_460_ value of the sensor also increased with increasing incubation duration for durations of 6 h and greater (Figure 3b). The change to the blue color became clearly apparent to the naked eye after an incubation of 11 h.

**Figure 4:**
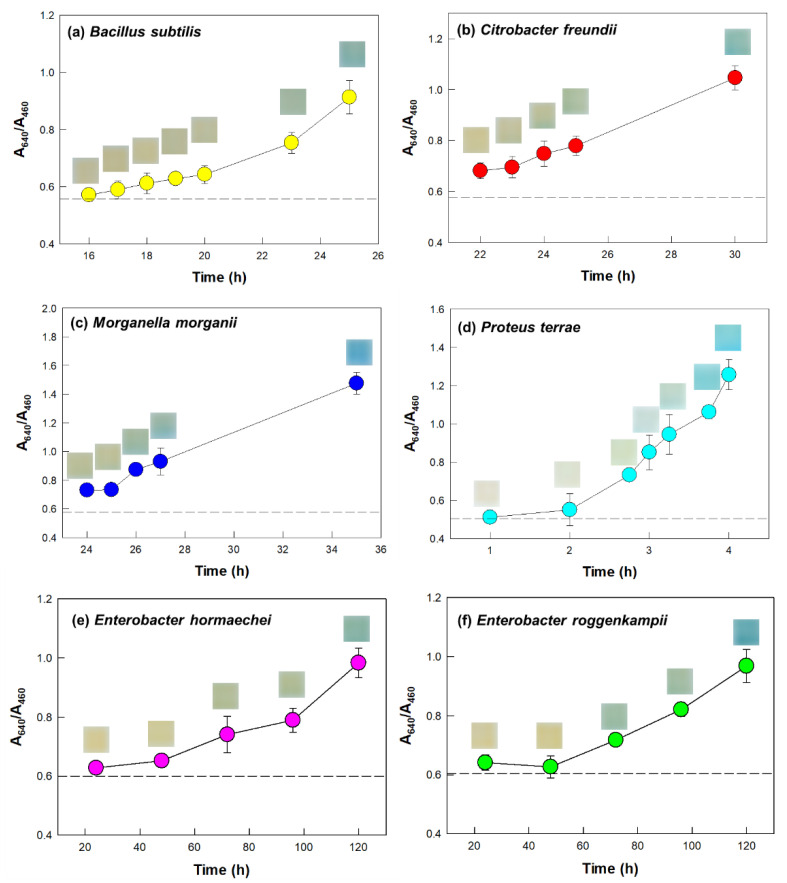
(**a**–**f**) Colorimetric detection of ureolytic bacteria using the designed film sensor. The A_640_/A_460_ values of sensors exposed to indicated bacteria plotted against incubation duration. Insets show photographs of the sensors. The dashed lines each indicate the A_640_/A_460_ value of the sensor in the absence of inoculum (negative control). Symbols and error bars represent the mean and standard deviations of biological triplicates.

The bacteria *Bacillus subtilis* (*B. subtilis*), *Citrobacter freundii* (*C. freundii*), *Morganella morganii* (*M. morganii*), *Klebsiella quasipneumoniae* (*K. quasipneumoniae*), and *Pseudomonas aeruginosa* (*P. aeruginosa*) showed moderately positive urease activities, and in each case, it took 23–26 h to achieve a recognizable change in the color of the sensor (Table 1, Figure 4a–c and Figure S1a,b). In each of these cases, the A_640_/A_460_ value of the sensor significantly increased after an incubation duration of ~24 h. The bacterium *P. terrae* showed strongly positive urease activity, as the color of the sensor exposed to this bacterium rapidly changed to blue within 3 h, and this change was clearly identified by the naked eye (Figure 4d). Meanwhile, the strains *Enterobacter hormaechei* (*E. hormaechei*) and *Enterobacter roggenkampii* (*E. roggenkampii*) showed weakly positive urease activities (Table 1, Figure 4e,f). In each of these cases, noticeable changes in color and A_640_/A_460_ of the sensor occurred only after about 3 days of reaction. When each of the bacterial strains *C. koseri*, *E. coli*, *K. michiganensis*, and *P. alcalifaciens* were tested, the sensor only marginally changed color after an extended reaction duration of 7 d (Table 1 and Figure S1c–f), indicating these four strains to be negative for urease.

Ureolytic activity has been described as a virulence factor for several bacteria and an emerging pathogenic factor during fungal infection [1,7,8]. The *Proteus* and *Klebsiella* species cause bacterial urinary tract infections in association with urease-dependent processes [7]. Urease-positive bacteria can result in the formation of infection stones and gastrointestinal colonization [26,27,28,29]. Infection stones surround the pathogens, thus protecting them. Moreover, ureolytic activity in human pathogens is thought to be related to the infectivity or persistence of the microbes [8].

Our above-described results indicate nine bacterial strains, namely *B. subtilis*, *C. freundii*, *E. hormaechei*, *E. roggenkampii*, *K. pneumoniae*, *K. quasipneumoniae*, *M. morganii*, *P. terrae*, and *P. aeruginosa*, to be urease-positive (but four other bacterial strains to be urease-negative), which is consistent with previous studies that also reported urease activity for these bacterial species [1,29,30,31,32,33,34,35]. *K. pneumoniae* is known as an opportunistic pathogen responsible for infections including pneumonia, septicemia, and urinary tract infections [26,27]. *Proteus* species display urease activity relevant to gastrointestinal pathogenicity [29]. *M. morganii* can cause sepsis, abscesses, purple urine bag syndrome, chorioamnionitis, and cellulitis [36]. Increasingly, *Klebsiella*, *Proteus*, and *Morganella* bacteria acquired antibiotic resistance, thereby posing a serious challenge for public health controls [29,36,37]. The results of our current study showed the possibility of employing our colorimetric sensor in a screening method for the identification of urease-producing pathogens.

### 3.4. Verification of the Detection of Urease Activity by the Sensor

**Figure 5:**
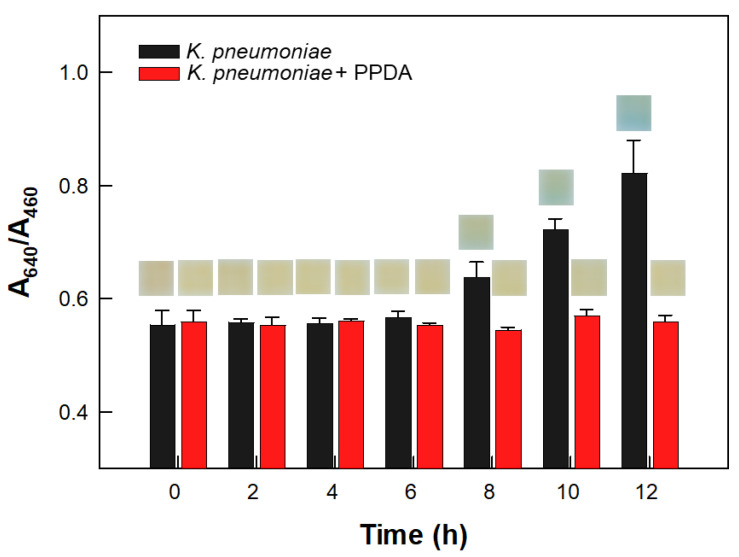
Verification of ureolytic activity in *Klebsiella pneumoniae* (*K. pneumoniae*) using a urease inhibitor, namely phenyl phosphorodiamidate (PPDA). The A_640_/A_460_ values and photographs (in insets showing colors) of film sensors in the absence and presence of PPDA are presented.

The colorimetric response of the film sensor to the ureolytic bacteria was verified using the urease inhibitor PPDA (Figure 5). The cells of the ureolytic bacterium *K. pneumoniae* not treated with PPDA (referred to non-treated cells) induced a change in the color of the sensor from yellow to blue within 10 h and achieved an increase in the A_640_/A_460_ value of the sensor as the reaction duration was increased (black bars of Figure 5). On the other hand, the sensor exposed to PPDA-treated cells (referred to *K. pneumoniae* + PPDA) showed neither any change in color nor in its A_640_/A_460_ value during 12 h of reaction (red bars of Figure 5). This lack of any significant colorimetric change was attributed to PPDA having specifically bound to the active site of urease, thus preventing not only the binding of urea to urease, but also the hydrolysis of urea to ammonia [4,6]. Based on these results, the colorimetric and absorbance spectral changes of the sensor exposed to non-treated cells were attributed to the reaction catalyzed by urease enzymes produced by *Klebsiella pneumoniae* cells.

A few studies have employed urease for the detection of bacteria [38,39,40]. Sun et al. (2018) employed urease-mediated three quaternized nanoparticle sensors for the detection of bacteria. The nanosensor was shown to be able to measure several Gram-positive and Gram-negative bacteria with an accuracy of 90.7% for 10^2^ CFU/mL within 30 min. However, it was incapable of achieving a selective detection of pathogenic bacteria [38]. Singh et al. (2019) employed silver–urease interactions for the colorimetric detection of pathogens [39]. Hou et al. (2020) used a 3D magnetic grid and urease catalysis for the rapid detection of *Salmonella* [40]. Instead of directly measuring bacterial urease activity, those studies used urease as a signal translation agent that catalytically produces ammonium carbonate, which could elevate the pH of the solution. The pH change was monitored by taking voltammetric measurements of the product of the catalyzed reaction (ammonium carbonate) or a pH-responsive chromogenic dye. Werkmeister et al. (2016) reported the use of organic field-effect transistors (OFETs) to sense ammonia for the purpose of detecting the enzymatically catalyzed breakdown of urea [41]. Due to the urease-catalyzed reaction, the OFETs responded to the millimolar levels of urea, but this feature did not extend to the detection of urease-producing microbes. According to our results, the developed colorimetric film sensor showed reliable performance for the detection of urease activity in various bacterial species.

### 3.5. Selective Detection of Urease-Producing Microbes in the Presence of Non-Ureolytic Bacteria

**Figure 6:**
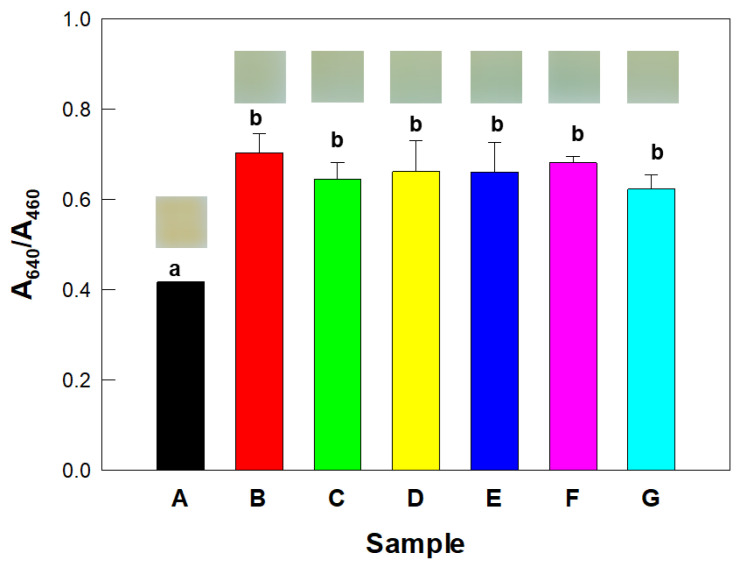
Colorimetric detection of urease activity of *Klebsiella pneumoniae* (*K. pneumoniae*) in the presence of non-ureolytic bacteria. The A_640_/A_460_ values and photographs of film sensors each acquired after an incubation duration of 10 h. A: *Citrobacter koseri* (*C. koseri*) + *Escherichia coli* (*E. coli*) + *Klebsiella michiganensis* (*K. michiganensis*) + *Providencia alcalifaciens* (*P. alcalifaciens*) (non-ureolytic bacteria only), B: *K. pneumoniae* only, C: *K. pneumoniae* + *C. koseri*, D: *K. pneumoniae* + *E. coli*, E: *K. pneumoniae* + *K. michiganensis*, F: *K. pneumoniae* + *P. alcalifaciens*, and G: *K. pneumoniae* + *C. koseri* + *E. coli* + *K. michiganensis* + *P. alcalifaciens*. Different lowercases indicate a statistical significance between the means of sample sets at a significance level (*p*-value) of 0.05.

The selectivity of the colorimetric film sensor for the detection of urease-producing microbes was validated using the ureolytic bacterium strain *K. pneumoniae*. An inoculum of only *K. pneumoniae* changed both the color and A_640_/A_460_ value of the sensor (Figure 6). That is, the sensor turned blue upon being exposed to an incubation of only *K. pneumoniae* cells (B in Figure 6) and comparable responses also occurred when *K. pneumoniae* cells were incubated with non-ureolytic bacteria (C–G in Figure 6). The presence of only non-ureolytic bacteria cells did not induce a change in the color of the sensor (A in Figure 6).

The photographic results were consistent with measurements of A_640_/A_460_ of the sensor. The A_640_/A_460_ value of the sensor increased only in the presence of *K. pneumoniae* cells (samples B–G in Figure 6) compared to that in the presence of only non-ureolytic bacterial cells (sample A in Figure 6), and these differences were statistically significant (*p* < 0.05, as determined from an ANOVA analysis). The non-ureolytic bacteria did not affect the A_640_/A_460_ value of the film sensor. That is, the A_640_/A_460_ values of the sensors exposed to *K. pneumoniae* cells with non-ureolytic bacteria (bars C–G of Figure 6) were statistically similar (*p* > 0.05) to that of the sensor exposed to only *K. pneumoniae* cells (sample B of Figure 6). As expected, the presence of only non-ureolytic bacterial cells induced just a marginal change in the A_640_/A_460_ value of the sensor (A in Figure 6).

### 3.6. Reusability of the Sensor

**Figure 7:**
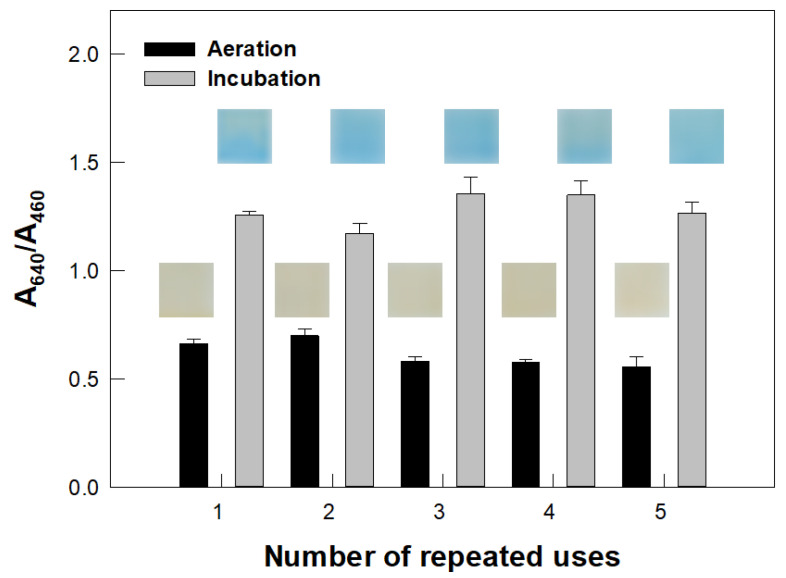
Reusability of the colorimetric film sensor. The A_640_/A_460_ values and photographs (showing colors) of a sensor reused five times are presented.

The color of a used sensor reverted from blue to its original yellow when subjected to aeration for 2 d (Figure 7). The sensor was found to be reusable for the detection of urease in *P. terrae* cells. Photographs and absorbance levels at 460 and 640 nm of the sensors were obtained between the repeated uses. The color of the sensor changed from yellow to blue upon the production of urease by *P. terrae* cells and the color change responses between the repeated uses were similar. Similarly, the A_640_/A_460_ value of the sensor increased after 4 h of incubation to between 1.17 ± 0.05 and 1.35 ± 0.08 between the repeated uses. The used sensor returned to its original state after each of five consecutive deployments.

The reusability of the sensor was attributable to the aeration having promoted the release of ammonia from the sensor (Figure 1d). The permeability of the PDMS layer allowed for the transport of ammonia gas into and out of the sensor, and thus the sensor was made reusable through simple aeration. Such reusability reduces fabrication and operation expenses, helping to make our sensor affordable.

## 4. Conclusions

We designed a colorimetric film sensor that could detect urease activity in microbes. Microbial urease activity was shown to induce a change in the color of the sensor from yellow to blue and this change in color can be simply identified by the naked eye. As a result of this color change property, the sensor can be used as an indicator of the presence of ureolytic microbes in a sample. The blue color development of the sensor allowed us to classify ureolytic microbes into strongly, moderately, and weakly positive urease activities by comparing the incubation times of thirteen different microbes. The sensor was found to be easy to use and to be selective and reusable for the detection of ureolytic microbes. We also expect its use to be extended to other applications, such as the monitoring of medical and environmental samples.

## Figures and Tables

**Table 1:** List of microbes used for the detection of urease activity.

Strain	Resource No.	Inoculum (CFU/mL)	Reaction Time (h)	Urease Activity ^a^
*Bacillus subtilis* subsp.	FBCC-B1550	1.5 ± 0.4 × 10^8^	23 h	++
*Citrobacter freundii*	FBCC-B1527	8.6 ± 2.0 × 10^8^	25 h	++
*Citrobacter koseri*	FBCC-B1520	2.2 ± 0.1 × 10^9^	−	−
*Enterobacter hormaechei* subsp.	FBCC-B414	7.7 ± 0.7 × 10^8^	72 h	+
*Enterobacter roggenkampii*	FBCC-B4	1.3 ± 0.2 × 10^9^	72 h	+
*Escherichia coli*	KCTC 2791	9.3 ± 4.0 × 10^8^	−	−
*Klebsiella michiganensis*	FBCC-B1517	6.2 ± 1.0 × 10^8^	−	−
*Klebsiella pneumoniae* subsp.	FBCC-B674	4.2 ± 2.0 × 10^8^	10 h	+++
*Klebsiella quasipneumoniae* subsp.	FBCC-B673	1.1 ± 0.1 × 10^9^	26 h	++
*Morganella morganii* subsp. *sibonii*	FBCC-B1534	1.1 ± 0.2 × 10^9^	26 h	++
*Proteus terrae*	FBCC-B448	1.4 ± 0.2 × 10^9^	3 h	+++
*Providencia alcalifaciens*	FBCC-B1524	2.2 ± 0.2 × 10^9^	−	−
*Pseudomonas aeruginosa*	FBCC-B567	9.5 ± 3.0 × 10^8^	24 h	++

^a^ −, negative; +, weakly positive; ++, moderately positive; and +++, strongly positive activity.

## Data Availability

Not applicable.

## Supplementary Materials

The following supporting information can be downloaded at: https://www.mdpi.com/article/10.3390/bios12100886/s1, Figure S1: (a–f) Colorimetric detection of ureolytic bacteria using the designed film sensor. A640/A460 values of sensors exposed to indicated bacteria plotted against incubation duration. Insets show photographs of the sensors. Symbols and error bars represent the mean and standard deviations of biological triplicates.
