# Supplementary material for: Colorimetric Detection of Urease-Producing Microbes Using an Ammonia-Responsive Flexible Film Sensor
Source: Biosensors (Basel). 2022 Oct 17;12(10):886. doi: 10.3390/bios12100886 (PMC9599750; doi:10.3390/bios12100886)
Supplement: Supplementary file 1 [file biosensors-12-00886-s001.zip › biosensors-1913525-supplementary.pdf]

[Supplementary information]

Colorimetric Detection of Urease-Producing Microbes Using an Ammonia-Responsive Flexible Film Sensor

Yunsoo Chang [1], Tae-Eon Park [2], Seung-Woo Lee [3,4,*] and Eun-Hee Lee [1,*]

[1] Department of Microbiology, Pusan National University, Busan 46241, Korea
[2] Center for Spintronics, Korea Institute of Science and Technology, Seoul 02792, Korea
[3] Department of Fine Chemistry, Seoul National University of Science and Technology, Seoul 01811, Korea
[4] Center for Functional Biomaterials, Seoul National University of Science and Technology, Seoul 01811, Korea
* Correspondence: swlee@seoultech.ac.kr (S.-W.L.); leeeh@pusan.ac.kr (E.-H.L.)

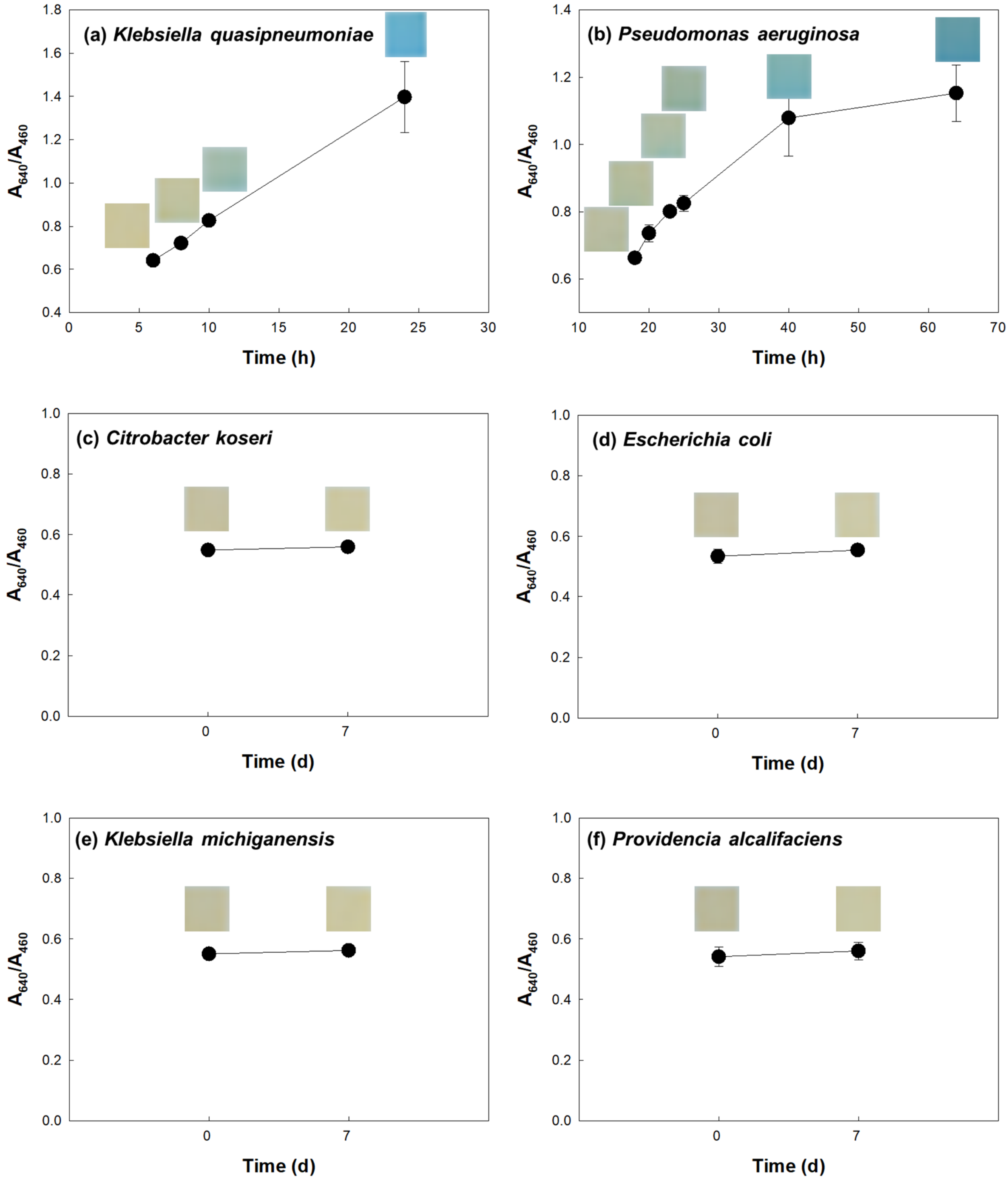

Figure S1. (**a–f**) Colorimetric detection of ureolytic bacteria using the designed film sensor. A_{640}/A_{460} values of sensors exposed to indicated bacteria plotted against incubation duration. Insets show photographs of the sensors. Symbols and error bars represent the mean and standard deviations of biological triplicates.
